# Electroacupuncture Attenuates Learning and Memory Impairment via PI3K/Akt Pathway in an Amyloid *β*_25-35_-Induced Alzheimer's Disease Mouse Model

**DOI:** 10.1155/2022/3849441

**Published:** 2022-04-15

**Authors:** Si-Mai Shao, Kyung Hye Park, Ye Yuan, Zijuan Zhang, Yanwen You, Zhenqiang Zhang, Li Hao

**Affiliations:** ^1^Medical College, Henan University of Chinese Medicine, Zhengzhou, China; ^2^Academy of Chinese Medical Sciences, Henan University of Chinese Medicine, Zhengzhou, China

## Abstract

The main characteristic of Alzheimer's disease (AD) is the progressive decline of learning and memory ability. Electroacupuncture (EA) may improve AD-related learning and memory ability. However, the underlying molecular mechanism of action remains unclear. The objective of the present study was to assess the effects and the molecular mechanism of EA on learning and memory in an amyloid *β*_25-35_ (A*β*_25-35_) induced AD mouse model. The AD model was established by intracerebroventricular (ICV) administration of A*β*_25-35_ oligomers. AD mice were electroacupunctured with wisdom three-needle combined with Baihui (GV20) five times per week for three consecutive weeks. The Morris water maze (MWM) and Y maze tests were applied to evaluate spatial learning and memory ability. A transmission electron microscope (TEM) was used to measure mitochondria and autophagy of hippocampal neurons, and western blot was applied to observe molecular changes in the mice hippocampus. The results suggested that EA treatment significantly alleviated learning and memory impairment related to AD, reduced mitochondria damage, improved autophagy, increased mitochondrial protein 2 (Mfn2), Beclin 1, and LC3B, and decreased the expressions of fission protein 1 (Fis1) level. Furthermore, EA further upregulated the protein expression of phosphatidylinositol 3-kinase (PI3K) and the ratio of p-Akt/Akt in the hippocampus of AD mice. This study demonstrates that EA treatment attenuates cognitive deficits, modulates mitochondrial fusion and fission, and enhances autophagy via the PI3K/Akt pathway in a mouse AD model.

## 1. Introduction

Alzheimer's disease (AD), the most common type of dementia, is one of the most prevalent neurodegenerative diseases affecting millions of older adults globally and is characterized by amyloid *β* (A*β*) deposition, phosphorylated tau accumulation, neuroinflammation, oxidative stress, and mitochondrial dysfunction [1–3]. Among them, A*β* deposition and phosphorylated tau accumulation are widely studied and considered the main pathological features of AD. In recent years, mitochondrial dysfunction has gradually gained attention and is considered to be closely related to various injuries in AD [4].

Studies suggest that mitochondrial damage and dysfunction are early pathological changes in AD and may precede the development of A*β* and tau pathology [5, 6]. Impaired mitochondrial function leads to inadequate bioenergy production, increased reactive oxygen species, and oxidative stress, which exacerbate A*β* deposition and tau protein phosphorylation, subsequently causing abnormal synaptic function and cognitive impairment [3]. Mitochondrial dysfunction is caused by abnormal mitochondrial dynamics, energy metabolism, and transport. Mitochondrial dynamics means the equalization between the two opposite processes of fusion and fission, which regulates mitochondria's number, morphology, and function in the cytoplasm [7]. Fusion helps to homogenize the composition of damaged mitochondria, thereby elongating the mitochondria. Fission leads to mitochondrial fragmentation [8]. Studies have confirmed that the accumulation of oligomeric A*β* in neurons of AD mice can cause increased mitochondrial fission, reduced fusion, mitochondrial and synaptic defects, and ultimately neurodegeneration [9, 10]. Impaired balance of mitochondrial dynamics in an AD mouse model has been associated with increased mitochondrial fragmentation [11]. Once the mitochondria are fragmented, the mitochondria's ability to produce ATP will be compromised and then it will be cleared.

Autophagy is an intracellular degradation process that degrades and recycles damaged organelles and proteins within the cell to maintain normal cellular homeostasis [12]. Therefore, autophagy is involved in clearing intracellular fragmented mitochondria, which is vital for regulating the mitochondrial number and maintaining physiological functions. Enhanced autophagy reduced AD-related tau hyperphosphorylation in neurons and reversed memory impairment in transgenic mice [13]. When autophagy is blocked, dysfunction of mitochondria transport and dynamics occurs in neurons, aggravating pathological changes in AD. Experiments have revealed that, in the early stages of AD, autophagy levels and the expression of Beclin 1 and LC3 are reduced and phosphatidylinositol 3-kinase (PI3K)/Akt pathway is also altered [14]. The PI3K/Akt pathway significantly mediates cellular autophagy and survival functions [15]. Increased autophagy and enhanced PI3K/Akt signaling in the brain can improve memory in AD mice [16]. It has been shown that induction of autophagy via the PI3K/Akt/mTOR pathway can reduce A*β* deposition in neurons [17]. Acupuncture improves autophagy in the brains of double transgenic AD mice, which may be associated with the PI3K/Akt pathway [18].

Acupuncture, an essential part of Chinese medicine, has progressed in clinical treatment and basic research on AD. Acupuncture can ameliorate cognitive deficits in patients with AD [19]. Previous studies have suggested that EA could upregulate enzymes involved in A*β* clearance, reduce the deposition of A*β*, protect hippocampal neurons, and improve AD-related learning and memory ability [20]. Wisdom three-needle is an acupuncture method that includes Shenting and Benshen and has been used clinically in China to treat neurodegenerative diseases such as dementia and stroke [21]. However, its molecular mechanism has not been clarified. Therefore, the aim of this study was to evaluate the molecular mechanism of wisdom three-needle combined with Baihui (GV20) electroacupuncture (EA) to improve the cognitive and mitochondrial dynamics in an amyloid *β*_25-35_ (A*β*_25-35_) induced AD mouse model.

## 2. Materials and Methods

### 2.1. Animals

Two-month-old male mice of C57Bl/6J (the classic standard laboratory mouse strain) were purchased from the Animal Experiment Center of Henan University of Chinese Medicine (Zhengzhou, China) (license number: SYXK (HA) 2020–0004). The mice were housed up to 3 per cage under laboratory animal ventilation system; the standard conditions are 12 h light/dark cycle, 22 ± 2°C controlled ambient temperature, and 50%–60% relative humidity, with ad libitum free access to food and water. All procedures involving animals and collection of tissue used in this study were in accordance with the “National Institutes of Health (NIH) Guide for the Care and Use of Laboratory Animals” and were approved by the Animal Welfare Committee of the Henan University of Chinese Medicine (approval number: DWLL201907311).

Forty mice were randomly assigned to four separate groups (*n* = 10/each): (1) the mice in the control group received bilateral hippocampal stereotactic microinjection of saline; (2) the mice in the model group received bilateral hippocampal stereotactic microinjection of A*β*_25–35_; (3) the mice in the EA group received wisdom three-needle combined with Baihui EA treatment after A*β*_25–35_ injection; (4) the mice in the nonacup group received EA at nonacupoints (0.5 cm above the root of the tail and 0.3 cm beside the midline) after A*β*_25–35_ injection.

### 2.2. Stereotactic Injection

**Figure 1:**
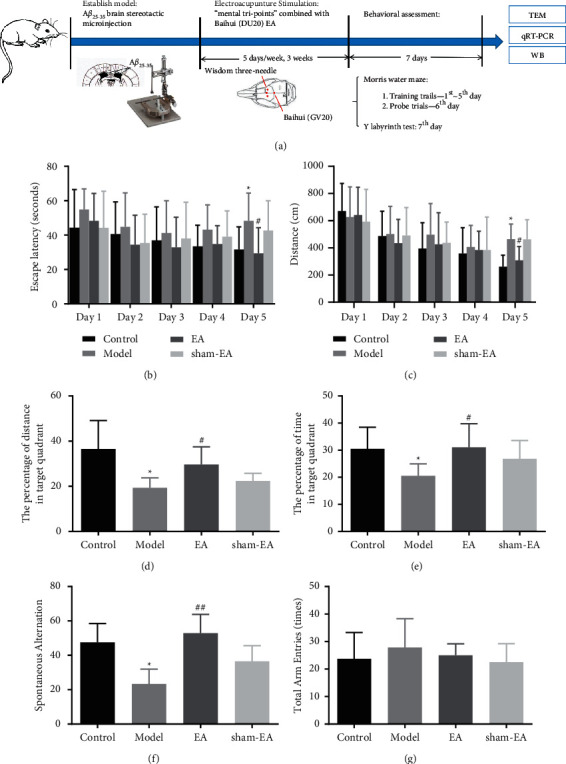
Behavioral test analysis of MWM test and Y-maze test has demonstrated that EA treatment could ameliorate learning and memory deficits in the AD mouse model. (a) The schematic diagram of the experimental design for this study. Mice were administered wisdom three-needle combined with Baihui EA therapy after bilateral hippocampal A*β*_25-35_ injections. Hippocampal tissue was collected after the last EA treatment. (b, c) The escape latency and total distance to reach the hidden platform were used to assess acquisition of the training trials in the MWM test. EA therapy shortened escape latency and total swimming distance in AD model mice on day 5. (d, e) The percentage of distance and time spent in target quadrant is shown which was calculated for probe trials in the MWM task. EA administration decreased the percentage of swimming distance and the percentage of time in the target quadrant in AD model mice on day 6. (f, g) The percentage of alternative behavior and the number of times of total arm entries as a measure of Y-maze test. EA significantly enhanced spontaneous alternation in AD mice. Data are represented as mean ± SD. ^*∗*^*P* < 0.05 vs. the control group; ^#^*P* < 0.05 and ^##^*P* < 0.01 vs. the model group.

A*β*_25-35_ (118M4893V; Sigma-Aldrich, MO, USA) was dissolved in 0.9% saline to a concentration of 3 mg/mL and incubated at 37°C for 7 days to induce aggregation [22]. After isoflurane anesthesia, the mice were fixed on the brain stereotactic apparatus, and the bilateral lateral ventricle (0.6 mm behind the anterior fontanel, 1.5 mm beside the midline, and 1.7 mm depth) was chosen as the site of microinjection. A*β*_25-35_ was injected into the lateral ventricles of mice in the model, EA, and sham-EA groups using a microsyringe at 3.5 *μ*L per side, and the injection was fully completed within 15 min. Mice in the control group were injected with equal sterile saline in the lateral ventricles (Figure 1(a)). No animals died during experimental injection and behavioral testing.

### 2.3. Electroacupuncture Treatment

Acupuncture points used in this study were according to the World Health Organization Standard Acupuncture Nomenclature. Baihui (GV20) is located at the midpoint of the parietal bone. Shenting (GV24) is 0.5 cm in front of Baihui. Benshen (GB13) is 0.3 cm beside Shenting. EA treatment was administered at 9 : 00 each day for 15 min, five days each week for three consecutive weeks. The disposable sterile needles were connected to the EA device's output terminals (G6805; Suzhou Medical Instrument Factory, Suzhou, China) using a 1∼20 Hz discrete wave and electric current of 1 mA. Mice in the EA group were treated with 0.30 × 13 mm needles (Suzhou Medical and Health Material Co., Ltd., Suzhou, China), with a 2-3 mm depth at the GV20, GV24, and GB13 points. Mice in the nonacup group were acupunctured at 0.5 cm above the root of the tail and 0.3 cm beside the midline (Figure 1(a)). They were also connected to an EA instrument but not energized.

### 2.4. Morris Water Maze (MWM) Test

To assess spatial cognitive performance in mice, the MWM (RWD Life Science Co., Ltd., Shenzhen, China) test was conducted from day 1 to day 6 after A*β*_25-35_ and EA administration. The experimental apparatus consisted of a circular tank with a diameter of 130 cm, a height of 50 cm, and an escape platform with a diameter of 10 cm. The tank was conceptually divided into four quadrants and filled with water (22 ± 2°C) made opaque by the milk powder. The platform was circular and hidden 1.5 cm below the surface. MWM was equipped with an infrared tracking camera computer and SMART 3.0 software (Panlab SL, Barcelona, Spain). The MWM test included training trial and probe trial.

  Training trial: each mouse was tested four times per day for five consecutive days. The platform was hidden in the same quadrant. During training, the mice were randomly placed in the water facing the wall of the tank and permitted to search the hidden platform for 60 seconds. If the mice failed to find the platform within 60 seconds, they were directed to the platform and maintained for 15 seconds. Escape latency (the time for mice to find the hidden platform) and total distance were recorded and analyzed.
  Probe trial: the probe trial was conducted on day 6. At the end of the training test, the platform was removed. The percentage of distance and time in the target quadrant (where the platform was located during the training trial) was recorded and analyzed. Ten mice per group participated in the MWM test.

### 2.5. Y-Maze Test

The Y-maze (RWD Life Technology Co., Ltd., Shenzhen, China) test was performed on the 7th day after A*β*_25-35_ and EA administration in this study (Figure 1(a)). The Y-maze consisted of three arms of equal length, and the angle between adjacent arms was 120. Mice were positioned in the middle of the Y-maze with the head facing the same arm and allowed to explore for 5 minutes. The SMART 3.0 software was employed to record the total times of arms entered. The number of alternations of the three arms accessed in the sequence divided by the alternation (total number of arms accessed minus 2) multiplied by 100 was the spontaneous alternation. Ten mice per group participated in this test.

### 2.6. Sample Preparation

Mice were executed after behavioral testing. The whole brains were removed after anesthesia with 20% (w/v) urethane, and the bilateral hippocampi were dissected on ice immediately and stored at −80°C for subsequent mRNA and protein assays, respectively. In addition, the hearts were perfused with precooled 4% paraformaldehyde (Damao Chemical Factory, Tianjin, China) and glutaraldehyde (Aladdin Industrial Co., Ltd., Shanghai, China). The CA1 region of the hippocampus was removed and quickly cut into small 1 mm^3^ tissue slices and placed in 4% glutaraldehyde fixative for transmission electron microscopy (TEM) analysis.

### 2.7. Transmission Electron Microscope

TEM was applied to investigate the mitochondrial ultrastructure and autophagy of hippocampal neurons. After sample preparation, the tissues were cut into 1 mm^3^ tissue blocks, postfixed in 2.5% paraformaldehyde solution for more than 2 h at room temperature, then rinsed with 0.1 M phosphoric acid (PBS) (P1010; Solarbio Life Sciences, Beijing, China) for 45 min, and then fixed in 1% osmic acid for 2 h at room temperature. After that, graded dehydration takes place for 20 min for each step in ethanol from water through 30%-50%-70%-85%–95% ethanol and twice for 30 min in 100% ethanol, followed by twice for 20 min in 50% ethanol/50% acetone (1 : 1) and twice for 20 min in 100% pure acetone. For embedding, tissues were incubated in pure acetone and embedding solution (2 : 1) at room temperature for 3 h, in pure acetone and embedding solution (1 : 2) at room temperature overnight, and in pure embedding solution at 37°C for 3 h. Tissues were dried at 37°C overnight, 45°C for 12 h, and 60°C for 24 h. Ultrathin 70 nm sections were obtained using an ultrathin sectioning machine and collected on 100-mesh copper grids. After staining with uranyl acetate and lead citrate (Head Bio Co., Ltd., Beijing, China), mitochondrial substructures and neuronal autophagy were observed on the sections by TEM (JEM-1400; Japan Electronics Co., Ltd, Japan). TEM analysis was performed by the Electron Microscopy Center of the Academy of Chinese Medicine, Henan University of Chinese Medicine. Three mice per group participated in this assay.

### 2.8. Quantitative Real-Time Polymerase Chain Reaction (qRT-PCR)

The mRNA levels of Mfn2, Fis1, Beclin 1, and LC3B were measured by qRT-PCR. Total RNA was extracted from the hippocampus with TRIzol reagent (15596026; Invitrogen, Thermo Fisher Scientific, MA, USA). The concentration of total RNA was measured with an ultraviolet spectrophotometer (ES-A01-NanoDrop 2000/2000c; Thermo Fisher Scientific, MA, USA). cDNA was synthesized using the PrimeScript™ RT reagent kit (RR037; Takara Biotechnology, Dalian, China). The ABI7500 system (Applied Biosystems, Foster City, CA, USA) was used to quantify the mRNA levels of Mfn2, Fis1, Beclin 1, and LC3B using the TB Green Premix Ex Taq kit (RR420; Takara Biotechnology, Dalian, China). The values were standardized to GAPDH. Data were analyzed with the 2^−ΔΔCt^ (cycle threshold, Ct) approach. The PCR primers (Sangon Biotech, Shanghai, China) were as follows Table 1. Five mice from each group attended the measurement.

### 2.9. Western Blot

Western blot was executed as indicated previously [23]. After sample preparation, frozen hippocampi were homogenized with tissue lysis buffer (R0010; Solarbio Life Science, Beijing, China) until lysis was complete. The supernatant was extracted following centrifugation. Moreover, the protein was quantified by using the BCA assay kit (Cat # PC0020; Solarbio Life Science, Beijing, China). The equal volume SDS-PAGE sample buffer (P1040; Solarbio Life Science, Beijing, China) was mixed into the protein supernatant and boiled at 100°C for 5 min until denaturation. Proteins were separated by polyacrylamide gel electrophoresis and transferred to a nitrocellulose membrane (IPVH00010, size 0.45 *μ*m; Merck Millipore Ltd., MA, USA). The membranes were blocked with 5% nonfat milk and then incubated with primary antibodies Fis1 (1 : 1000, ab96764; Abcam, Cambridge, UK), Mfn2 (1 : 1000, ab124773; Abcam, Cambridge, UK), LC3B (1 : 1000, ab48394; Abcam, Cambridge, UK), Beclin 1 (1 : 2000, ab207612; Abcam, Cambridge, UK), PI3K (1 : 1000, ab139307; Abcam, Cambridge, UK), Akt (1 : 1000, ab179463; Abcam, Cambridge, UK), p-Akt [S473P] (1 : 1000, ab81283; Abcam, Cambridge, UK), and *β*-actin (1 : 2000, ab8226; Abcam, Cambridge, UK) at 4°C overnight. Afterward, the membrane was incubated with horseradish peroxidase (HRP) conjugated goat anti-rabbit IgG and goat anti-rabbit IgG (1 : 5000, Boster, Wuhan, China) for 1 h. The proteins were measured by ECL chemiluminescence (VJ312149; Thermo Fisher Scientific, Massachusetts, USA). Quantification of all bands was performed using Image J v1.51 (National Institutes of Health, Bethesda, Maryland). Three mice per group participated in this assay.

### 2.10. Statistical Analysis

The results obtained from the experiments were expressed as mean ± standard deviation (SD). Statistical significance differences were assessed with one-way analysis of variance (ANOVA) followed by a Bonferroni's post hoc test. Behavioral tests from day 1 to day 5 of the MWM were analyzed by two-way ANOVA. Statistical analyses and figures were based on GraphPad Prism 8.0 software (GraphPad Software, CA, USA). *P* values less than 0.05 were considered statistically significant.

## 3. Results

### 3.1. EA Ameliorated Learning and Memory Deficits in an AD Mouse Model

The MWM test was used to detect the effect of wisdom three-needle combined with Baihui (GV20) EA on AD mice's cognitive abilities. In the training trials, there was no significant difference in escape latency and total swimming distance among four groups from day 1 to day 4. However, the escape latency and total swimming distance of the model group were significantly longer than those of the control group on day 5 (*P* < 0.05), and the escape latency and total swimming distance of the EA group were significantly shorter than those of the model group (*P* < 0.05). In addition, there was no significant difference between AD model group and sham-EA group (Figures 1(b) and 1(c)). In the probe trials, the percentage of swimming distance and the percentage of time in the target quadrant of the model group were less than those of the control group (*P* < 0.05). Moreover, the percentage of swimming distance and the percentage of time in the target quadrant of the EA group were more than those of the model group (*P* < 0.05). Furthermore, there was still no statistical difference between the model and the sham-EA groups (Figures 1(d) and 1(e)).

The Y-maze test was carried out to test mice's spatial working and reference memory. Spontaneous alternation was assessed by exploring the three arms of the maze in mice. The spontaneous alternation of the model group was less than that of the control group (*P* < 0.05), and the spontaneous alternation of the EA group was more than that of the model group (*P* < 0.01) (Figure 1(f)). However, there were no differences in total entrances among the four groups (Figure 1(g)).

In general, behavioral data showed that wisdom three-needle combined with Baihui (GV20) EA could improve AD-related learning and memory deficits.

### 3.2. EA Regulated Mitochondrial Fusion/Fission Dynamics in an AD Mouse Model

**Figure 2:**
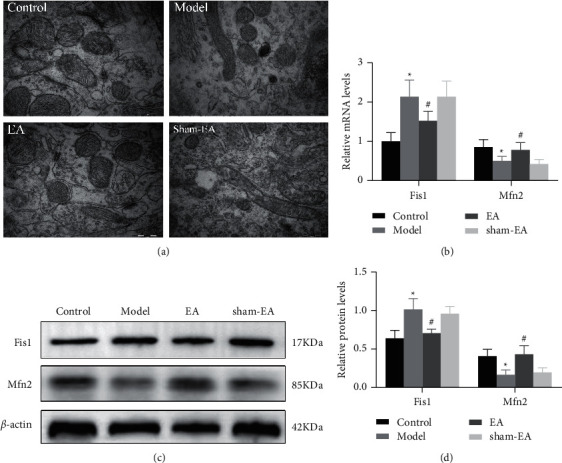
Transmission electron microscope and western bolt results showed that EA treatment modulated mitochondrial fusion/fission dynamics in AD mouse models. (a) TEM detected the mitochondrial structure of hippocampal neurons in each group in the CA1 regions of mice brain. Scale bar in (a) = 500 nm (magnification = 50000x). EA therapy improved mitochondrial morphology of neurons in the hippocampus of AD mice. (b) Mitochondrial dynamics correlation genes Fis1 and Mfn2 in the hippocampus of the mouse were detected by qRT-PCR. EA downregulated Fis1 and upregulated Mfn2 mRNA levels, respectively. (c, d) Protein levels of Fis1 and Mfn2 in the mouse hippocampus were measured by quantitative immunoblot analysis. *β*-actin was used as a loading control. EA administration decreased Fis1 and increased Mfn2 protein expression, respectively. The data (*n* = 3) are represented as mean ± SD. ^*∗*^*P* < 0.05 vs. the control group; ^#^*P* < 0.05 vs. the model group.

Studies have confirmed that cognitive deficits in AD were accompanied by changes in the mitochondrial dynamics of hippocampal neurons [24]. Therefore, we hypothesized that EA treatment reduced cognitive deficits by regulating mitochondrial dynamics. To test this hypothesis, we evaluated the mitochondrial morphology of hippocampal neurons of the mice in all groups by TEM. The results indicated that A*β*_25-35_ caused the morphological changes of mitochondria in hippocampal neurons of AD mice. It can be demonstrated by the decrease in the number of mitochondria, swelling and breakage of the cristae, and the rupture of the membrane. EA treatment improved mitochondrial morphology in the hippocampus of AD mice. There was no difference between the model group and the sham-EA group (Figure 2(a)).

To study the potential mechanism of EA treatment on improving mitochondrial morphology, we further measured some genes and proteins related to mitochondrial dynamics. It has been shown that Fis1 promotes mitochondrial fission [25] and the mRNA and protein expression of Fis1 was significantly increased in the hippocampus of AD mice compared to that of the control group (*P* < 0.05). EA treatment decreased the mRNA and protein levels of Fis1 in AD mice. Mfn2 contributes to mitochondrial fusion, and the mRNA and protein levels of Mfn2 in the hippocampus of AD mice decreased compared with those of the control group (*P* < 0.05), whereas EA treatment significantly increased its mRNA and protein levels (Figures 2(b)–2(d)). There was no difference in Fis1 and Mfn2 between the control group and the sham-EA group. These results suggested that EA treatment reduced mitochondrial fission and promoted mitochondrial fusion, which in turn regulates mitochondrial dynamics.

### 3.3. EA Improved Autophagy in an AD Mouse Model

**Figure 3:**
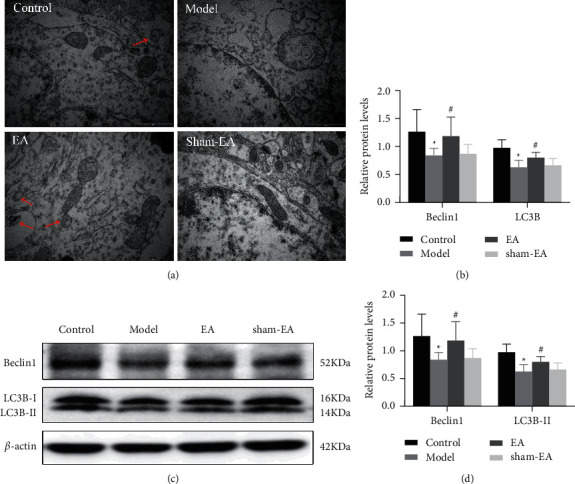
Transmission electron microscope and western bolt results showed that EA treatment improved autophagy in AD mouse models. (a) TEM observation of autophagosome in the CA1 regions of mice brains in each group. EA therapy resulted in an increase in the number of autophagosomes in hippocampal neurons of AD mice. Scale bar in (a) = 1 *μ*m (magnification = 20000x). (b) Autophagy-related genes Beclin 1 and LC3B in the hippocampus of the mouse were measured by qRT-PCR. EA treatment enhanced the mRNA levels of Beclin 1 and LC3B. (c, d) Beclin 1 and LC3B-II in hippocampus of the mouse were detected by immunoblotting quantitative analysis. *β*-actin was used as a loading control. EA administration elevated the protein expression of Beclin 1 and LC3B-II. The data (*n* = 3) are represented as mean ± SD.^*∗*^*P* < 0.05 vs. the control group; ^#^*P* < 0.05 vs. the model group.

In the pathogenesis of AD, abnormal autophagy can cause mitochondrial dysfunction, reduced ATP synthesis, and energy metabolism inhibition, leading to neuronal cell loss [26]. To investigate the reason of mitochondrial dynamics dysfunction, autophagy was examined by transmission electron microscopy. Autophagosome is the primary basis to evaluate the appearance of autophagy, which is characterized by double or multilayer vacuole structure. The TEM results showed decreased intracellular autophagosomes in hippocampal neurons in the AD model group compared with the control group and increased intracellular autophagosomes in hippocampal neurons in the EA group compared with the model group. Nevertheless, there was no difference between the model group and the sham-EA group (Figure 3(a)). Beclin 1 is a critical protein in the formation of autophagosomes. During autophagy, LC3B-I is modified and processed by the ubiquitin system, producing small molecules of LC3B-II that are localized into autophagosomes. Thus, both the presence of LC3B and high levels of LC3B-II are considered molecular markers of autophagy in cells undergoing ferroptosis [27]. We found that AD model mice had a significantly decreased mRNA expression of Beclin 1 and LC3B and protein levels of Beclin 1 and LC3B-II compared with that of control mice (*P* < 0.05) (Figures 3(b)–3(d)). Wisdom three-needle combined with Baihui (GV20) EA treatment significantly increased the mRNA expression of Beclin 1 and LC3B and the protein levels of Beclin 1 and LC3B-II (*P* < 0.05). These results indicated that EA treatment enhanced neuronal autophagy, contributing to the improved learning and memory impairment in AD mice.

### 3.4. EA Ameliorated Autophagy through PI3K/Akt Pathway

**Figure 4:**
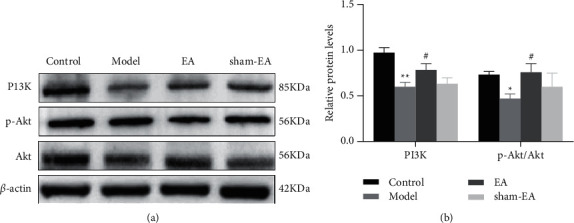
Western bolt results showed that EA treatment regulated the PI3K/Akt pathway. (a, b) The protein levels of PI3K, Akt, and p-Akt in the hippocampus of the mouse measured by western blot analysis. *β*-actin was used as a loading control. The data (*n* = 3) are represented as mean ± SD.^*∗*^*P* < 0.05 and ^∗∗^*P* < 0.01 vs. the control group; ^#^*P* < 0.05 vs. the model group.

The PI3K/Akt pathway was found to play an essential role in regulating neuronal autophagy [28]. To further elucidate the underlying molecular mechanisms of EA improving autophagy, we detected the expression level of PI3K, Akt, and its activated form p-Akt (Ser473) in the hippocampus of AD model mice. The expression levels of PI3K and p-Akt/Akt were significantly lower in AD mice, and these changes were restored partly by wisdom three-needle combined with Baihui (GV20) EA treatment. However, no changes were observed in the sham-EA group (Figures 4(a)–4(c)). These results suggested that EA treatment has a specific ameliorative effect on autophagy, which may be related to the downregulation of PI3K and p-Akt/Akt.

## 4. Discussion

In the present study, we identified the neuroprotective role of wisdom three-needle combined with Baihui (GV20) EA treatment, which attenuated mitochondrial damage and enhanced neuronal autophagy in AD model mice and its mechanism of this protection. In addition, we observed a neuroprotective role of the PI3k/Akt pathway in EA treatment of A*β*_25-35_-induced cognitive impairment.

MWM and Y-maze play an essential role in validating animal models of cognitive impairment such as AD and aging [29]. In this investigation, wisdom three-needle combined with Baihui (GV20) EA treatment reduced the escape latency and upregulated the percentage of swimming distance and residence time in the target quadrant compared with AD mice. Simultaneously, we found that EA could improve spontaneous alternation behavior in AD mice. Therefore, the behavior test results suggested that wisdom three-needle combined with Baihui (GV20) EA treatment enhanced the learning and memory abilities in AD mice.

Consistent with the behavioral test results, we revealed that wisdom three-needle combined with Baihui (GV20) EA treatment attenuated mitochondrial damage. In the hippocampus neurons of A*β*_25-35_-induced mice, damaged mitochondria with an indistinct morphology were observed. Notably, this type of damaged mitochondria was not observed in mice treated with EA. Mitochondrial dysfunction and accumulated damaged mitochondria are prevalent in AD patients and transgenic AD model animals. It has been demonstrated that A*β* aggregation caused an imbalance of mitochondrial fission and fusion, mitochondrial fragmentation, and morphological and functional changes, leading to mitochondrial damage [30]. Fis1 and Mfn2 are closely associated with mitochondrial fission and fusion, respectively. The mRNA and protein levels of Fis1 and Mfn2 decreased and increased with EA treatment, suggesting that EA can modulate mitochondrial dynamics.

The primary way to eliminate damaged mitochondria and mitochondrial fragmentation is autophagy in AD [31]. Autophagy, the selective removal of damaged mitochondria by autophagosomes followed by catabolism by lysosomes, is essential for maintaining mitochondrial homeostasis, ATP production, and neuronal survival [32]. LC3B-II is considered as a marker molecule in autophagosomes [33]. It was found that the expression of LC3B-II and Beclin 1 in the AD brain was low, which resulted in decreased autophagy and A*β* accumulation in the brain of the transgenic mouse model [34]. In this study, EA administration also increased the mRNA expression of Beclin 1 and LC3B and the protein levels of Beclin 1 and LC3B-II in AD model mice, indicating that EA can enhance neuronal autophagy.

The PI3K/Akt signaling pathway has been found to regulate neuronal autophagy, and its deregulation results in decreased autophagy. Activation of PI3K/Akt prevents neuronal cell death in AD; moreover, activation of PI3K/Akt in neurons has been reported as neuroprotective in AD rats [35]. Given that the PI3K/Akt pathway is involved in neuronal autophagy, survival, and synaptic plasticity, it has attracted much attention as a therapeutic target [36]. Therefore, we speculated that EA treatment improved abnormal mitochondrial dynamics and enhanced autophagy by activating the PI3K/Akt pathway in hippocampal neurons. This speculation underlines the potential beneficial effects of EA, as EA might activate the PI3K/Akt pathway when triggers such as mitochondrial damage and reduced levels of autophagy are encountered. As a critical molecule in the PI3K/Akt pathway, Akt governs the survival and loss of neurons [37]. Enhanced Akt activation facilitates increased autophagy and neuroprotective effects in the AD brain. Akt has two major phosphorylation sites, thr308 and Ser473, and the activation of Akt mainly requires phosphorylation of Ser473 [38]. In the current experiments, p-Akt (Ser473) expression was significantly reduced in the hippocampus of AD mice and EA reversed this reduction; however, Akt expression was not substantially altered, indicating that Akt exerts its function through phosphorylation rather than regulation.

In conclusion, our study sheds light on the potential of EA to attenuate cognitive deficits, modulate mitochondrial dynamics, and enhance autophagy in AD mice via the PI3K/Akt pathway. This result lays the experimental foundation for the application of wisdom three-needle combined with Baihui (GV20) to treat neurodegenerative diseases.

## Figures and Tables

**Table 1:** The primer sequences used in qRT-PCR.

Genes	Primer
Mfn2	Forward: 5′-TGCACCGCCATATAGAGGAAG-3′ Reverse: 5′-TCTGCAGTGAACTGGCAATG-3′
Fis1	Forward: 5ʹ-CAAAGAGGAACAGCGGGACT-3ʹ Reverse: 5ʹ-ACAGCCCTCGCACATACTTT-3ʹ
Beclin 1	Forward: 5ʹ-ATGGAGGGGTCTAAGGCGTC-3ʹ Reverse: 5ʹ-TGGGCTGTGGTAAGTAATGGA-3ʹ
LC3B	Forward: 5ʹ-GATAATCAGACGGCGCTT-3ʹ Reverse: 5ʹ-ACTTCGGAGATGGGAGTG-3ʹ
GAPDH	Forward: 5′-TTCCCGTTCAGCTCTGGG-3′ Reverse: 5′-CCCTGCATCCACTGGTGC-3′

## Data Availability

The experimental data supporting this study's findings are available from the corresponding authors upon reasonable request.
